# LY3214996 relieves acquired resistance to sorafenib in hepatocellular carcinoma cells

**DOI:** 10.7150/ijms.51256

**Published:** 2021-01-29

**Authors:** Yongfang Ma, Ruyue Xu, Xueke Liu, Yinci Zhang, Li Song, Shuyu Cai, Shuping Zhou, Yinghai Xie, Amin Li, Weiya Cao, Xiaolong Tang

**Affiliations:** 1 Medical school, Anhui University of Science and Technology, Huainan 232001, China.; 2 Institute of Environment-friendly Materials and Occupational Health of Anhui University of Science and Technology (Wuhu), Wuhu, 241003, China.; 3 Department of Clinical Laboratory Medicine, The Fourth Affiliated Hospital, Zhejiang University School of Medicine, Yiwu 322000, China.

**Keywords:** sorafenib, LY3214996, hepatocellular carcinoma, Ras/Raf/MAPK pathway, acquired resistance

## Abstract

**Background:** Sorafenib, an oral multi-kinase inhibitor of rapidly accelerated fibrosarcoma; vascular endothelial growth factor receptor-2/3, platelet-derived growth factor receptor, c-Kit, and Flt-3 signaling, is approved for treatment of advanced hepatocellular carcinoma (HCC). However, the benefit of sorafenib is often diminished because of acquired resistance through the reactivation of ERK signaling in sorafenib-resistant HCC cells. In this work, we investigated whether adding LY3214996, a selective ERK1/2 inhibitor, to sorafenib would increase the anti-tumor effectiveness of sorafenib to HCC cells.

**Methods:** The Huh7 cell line was used as a cell model for treatment with sorafenib, LY3214996, and their combination. Phosphorylation of the key kinases in the Ras/Raf/MAPK and PI3K/Akt pathways, protein expression of the cell cycle, and apoptosis migration were assessed with western blot. MTT and colony-formation assays were used to evaluate cell proliferation. Wound-healing assay was used to assess cell migration. Cell cycle and apoptosis analyses were conducted with flow cytometry.

**Results:** LY3214996 decreased phosphorylation of the Ras/Raf/MAPK and PI3K/Akt pathways, including p-c-Raf, p-P90RSK, p-S6K and p-eIF4EBP1 activated by sorafenib, despite increased p-ERK1/2 levels. LY3214996 increased the anti-proliferation, anti-migration, cell-cycle progression, and pro-apoptotic effects of sorafenib on Huh7^R^ cells.

**Conclusions:** Reactivation of ERK1/2 appears to be a molecular mechanism of acquired resistance of HCC to sorafenib. LY3214996 combined with sorafenib enhanced the anti-tumor effects of sorafenib in HCC. These findings form a theoretical basis for trial of LY3214996 combined with sorafenib as second-line treatment of sorafenib-resistant in advanced HCC.

## Introduction

Hepatocellular carcinoma (HCC) is the sixth commonest cancer and the fourth leading cause of cancer-related death in the world, as reported by the International Agency for Research on Cancer 1-2. HCC, which occurs usually in the setting of chronic liver disease and cirrhosis, makes up about 90% of all cases of primary liver cancer. The curative treatment options for HCC are surgical resection, liver transplantation, and local ablative therapies. Although these measures are effective for early-stage HCC, around 80% of cases are diagnosed in advanced stages when the treatment options are limited. Thus, the prognosis of advanced HCC is poor, with median overall survival time less than one year 3. Systemic therapy is a hopeful but unproven approach for extending the lives of HCC patients.

Sorafenib, an oral multi-target, multi-kinase inhibitor, is the most widely used systemic chemotherapy for HCC. It was approved as a first-line agent for unresectable or advanced disease by the United States Food and Drug Administration in 2007 4-5. On the one hand, sorafenib inhibits the receptor tyrosine kinases c-Kit and Flt-3 6 and the serine/threonine kinases, including Raf kinases involved in the Raf/MEK/ERK pathway 7, which leads to inhibition of tumor-cell proliferation. On the other hand, sorafenib inhibits the receptor tyrosine kinase, including vascular endothelial growth factor receptor-2/3 and platelet-derived growth factor receptor, which inhibits tumor angiogenesis 8. Despite encouraging progress in the treatment of HCC with sorafenib, response rates for patients with advanced disease are poor, largely because of acquired resistance to the drug. Other obstacles, including epithelial-mesenchymal transition, Ras/Raf/MAPK, PI3K/Akt, JAK/STAT pathways, and hypoxia also contribute to the failure of sorafenib in HCC patients 9-11. Hence, new treatment options for advanced HCC are needed.

Recently, aberrant rapidly accelerated fibrosarcoma (Raf) /mitogen-activated protein kinase, MEK/extracellular signal-regulated kinase, and ERK signaling pathway activation have been identified as central for cancer growth, motility, and survival. The Ras/Raf/MAPK cascade participates in cell-cycle regulation, apoptosis, and cell differentiation 15. The cascade also plays an important role in apoptosis by phosphorylating apoptosis-regulating factors, such as Bad, Bim, Mcl-1, caspase 9, and the controversial Bcl-2 16. Consequently, drugs have been developed to inhibit Ras/Raf/MAPK signals in cancer, and Raf and MEK inhibitors have been approved for clinical use 17-18. The combination of Raf /MEK inhibition seems to improve progression-free survival compared to survival with Raf or MEK monotherapy 19. Despite the success of these treatments, almost all HCC patients develop resistance to the drugs 20. Many studies on mechanisms of resistance to Raf/MEK inhibition have been described. The most common mechanisms activate MAPK pathways through multiple methods, including alternate splicing B-RAF, N-RAS, or MEK1/2 mutations, upregulation of MAPK38, clinical drug resistance mechanisms, and/or receptor tyrosine kinase signaling 21-22. Since the pathway from Raf to MEK to ERK is linear, ERK1/2 itself has been a target, and all these changes are focused on the continuous activation of ERK. Hence, the clinical development of small-molecule ERK inhibitors has aroused interest. LY3214996, a potent, selective, ATP-competitive ERK inhibitor, inhibits the pharmacodynamic biomarker, phospho-P90RSK, which may be the mechanism of its antitumor activities 23.

In this study, we investigated the mechanism of acquired resistance to sorafenib in HCC, and we combined LY3214996 with sorafenib to restore chemosensitivity of drug-resistant HCC cells. Our results provide theoretical basis for combination therapy of sorafenib and ERK1/2 inhibitors in treatment of HCC.

## Materials and methods

### Reagents and antibodies

Sorafenib was obtained from MedChem Express (Monmouth Junction, NJ, USA). ERK1/2 inhibitor (LY3214996) was purchased from Selleckchem (Houston, TX, USA). Antibodies to cyclin D1 and phospho-Rb were purchased from Abcam Biological Technology (USA). Phospho-ERK1/2 Kit (#9911) and antibodies to ERK1/2, Caspase-3, Cleaved Caspase-3, Caspase-9, Cleaved Caspase-9, PARP, Cleaved PARP, PI3K110β, Akt, phospho-Akt (Ser473), mTOR, phospho-mTOR (Ser2481), Bim, Bad, Bak, Bax, P70S6K, S6K, β-actin, phospho-S6K, phospho-P70S6K and secondary horseradish peroxidase (HRP)-conjugated goat anti-rabbit and anti-mouse antibodies were purchased from Cell Signal Technology (Danvers, MA, USA).

### Cell lines and cell culture

Huh7, a human HCC cell line, was purchased from American Type Culture Collection (Rockville, MD, USA). Huh7^R^, an acquired sorafenib-resistant HCC cell line, was established from Huh7 cells as follows: When the cells were in the logarithmic growth phase, the culture medium was changed, and a lower concentration of sorafenib was added for 24 h. Cell inheritance was performed, and stimulation with this concentration was repeated until it was stable. When the sorafenib concentration reached 4-5 times the IC_50_ value of the sensitive strain, the resistant strain was obtained. The two cell lines were cultured in RPMI-1640 (Hyclone, Salt Lake City, UT, USA), supplemented with 15% fetal bovine serum (Sijiqing Bioengineering Materials, Hangzhou, China) and incubated at 37 °C in 5% CO_2_.

### Cell viability assay

Cells (10,000 cells per well) were seeded into 96-well plates, allowed to adhere overnight, and exposed to a range of drug concentrations. After 12, 24, 48, and 72 h, 10 µl of 5 mg/ml MTT dissolved in PBS was added to each well and incubated for 4 h at 37 °C. The medium was aspirated, and 100 µl of dimethyl sulfoxide was added to each well and shaken for 15 min. Absorbance was measured at 490 nm. IC_50_ value was calculated by GraphPad Prism Version 5.0 software.

### Colony-formation assay

Cells were plated in six-well plates at a density of 2×10^3^ cells per well for 24 h, and drug experiments as previously designed were carried out. After two weeks, when cellular clones were visible, the cells were fixed in 4% paraformaldehyde for 15 min, and then stained with 0.1% crystal violet for 30 min. The 6-well plates were thoroughly washed with water for counting of the clones. The rate of colony formation reflects two important characteristics of cell population: dependence and proliferation ability. All experiments were done in triplicate.

### Wound-healing assay

Cells (2×10^5^ cells/well) were plated overnight in 6-well plates with 15% fetal bovine serum medium. Injured cells were removed by washing with PBS. Serum-free medium containing drugs was added to corresponding wells and incubated at 37 °C in a humidified atmosphere in 5% CO_2_. The width of the scratched area after 24 and 48 h was recorded by pictures taken with a light microscope.

### Cell-cycle assay

Cells were digested with 0.25% trypsin-EDTA after being incubated with various drug combinations for 24 h, washed with PBS three times, and fixed with 70% pre-cooled ethanol. Before testing, the cells were rewashed by PBS, 50 μg/ml propidium iodide, and 100 μg/ml RNase A were added and reacted in the dark for 30 min at 37 °C. Flow cytometry analysis was conducted (with BD FACSCalibur, USA); the distribution of cells at cell-cycle stages was assessed with ModFit Version 3.0 software (Verity Software House, Topsham, ME).

### Apoptosis assay

After drug treatment for 24 h, cells from the experimental groups were collected. The cells were washed with cold PBS, resuspended in 500 µL binding buffer containing 5 µl propidium iodide and 10 µl FITC-labelled Annexin-V, and incubated for 15 min in the dark at room temperature. Cell apoptosis was detected with Flow cytometry analysis (BD Biosciences), and apoptotic rate was measured with FlowJo Version 7.6.1 software (FlowJo, Ashland, OR).

### Western blot

Whole cell lysates were obtained with the combination of radio immunoprecipitation assay buffer (Beyotime, Jiangsu, China) and a mixture of protease and phosphatase inhibitor (Beyotime, Jiangsu, China). Protein concentration was measured with BCA protein assay kit (Biosharp, Hefei, China). Supernatants per sample were aliquoted, mixed with loading buffer, separated by sodium dodecyl sulfate-polyacrylamide gel electrophoresis (SDS-PAGE), and transferred to a polyvinylidene fluoride membrane. The membranes were blocked with 5% skim-milk, primary antibodies were applied and reacted at 4 °C for 24 h, and the secondary antibody was applied and reacted for 1 h at 37 °C. Visualization of the signals was clarified with Western ECL substrate (Bio-Rad, Hercules, CA). Protein expression was quantified with Image J Version 1.48 software (NIH, Bethesda, MD) with β-actin as a loading control.

### Statistical analysis

All *in vitro* assay results represent three replicates of three independent experiments performed under the same conditions. All data were expressed as mean values ± standard deviation. Statistical tests were performed on the paired values with Student's t-test; *p*<0.05 was considered statistically significant.

## Results

### PI3K/Akt and Ras/Raf/MAPK pathways were stably activated in Huh7^R^ cells

**Figure 1:**
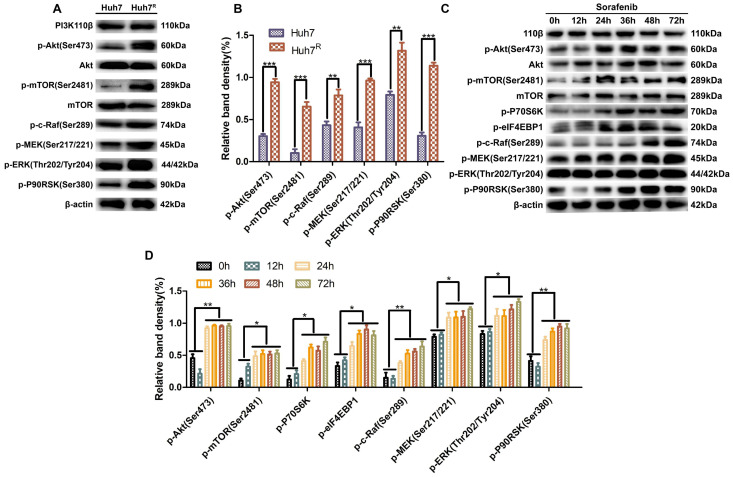
PI3K/Akt and Ras/Raf/MAPK pathways were stably activated in Huh7^R^ cells. (**A** and **B**) Major kinase levels of PI3K/Akt and Ras/Raf/MAPK pathways in Huh7 and Huh7^R^ cells were examined by western blot. (**C** and **D**) Kinase expression levels in related pathways in Huh7^R^ cells treated with sorafenib at various times were detected by western blot. Data expressed as mean ± SD of three independent experiments (*p<0.05; **p<0.01; ***p<0.001).

To learn the activation status of PI3K/Akt and Ras/Raf/MAPK pathways in Huh7^R^ and Huh7 cells, we analyzed the phosphorylation levels of the main kinases of the related pathways in the two cells lines. Western blot results revealed that the expression of p-Akt (Ser473), p-mTOR (Ser2481), p-S6K, p-eIF4EBP1, p-c-Raf (Ser289), p-MEK (Ser217/221), p-ERK (Thr202/Tyr204), and p-P90RSK (Ser380) in Huh7^R^ cells was higher than in Huh7 cells (Fig. 1A and B), a result that was evidence that the above pathways were activated in Huh7^R^ cells. Next, Huh7^R^ cells were treated with sorafenib for 0 h, 12 h, 24 h, 36 h, 48 h, and 72 h at the same concentration to assess for the activated form of the two pathways. Western blot results showed that the phosphorylation levels of major kinases in the two pathways were decreased at 12 h compared with 0 h, reached the lowest level at 24 h, and were in stable activation from 24 h to 72 h (Fig. 1C and D).

### LY3214996 enhanced sorafenib anti-proliferative effects in Huh7^R^ cells

**Figure 2:**
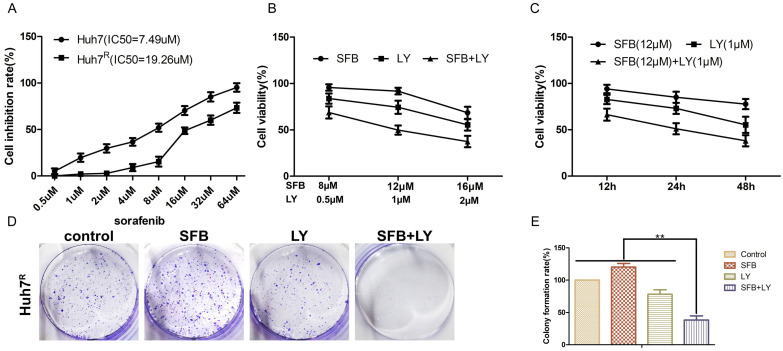
LY3214996 enhanced sorafenib anti-proliferative effects in Huh7^R^ cells. (**A**) MTT assays detected cell viability of Huh7 and Huh7^R^ cells incubated with various concentrations of sorafenib for 24 h. (**B**) Cell viability of Huh7^R^ cells treated with sorafenib, LY3214996, and the combination after 24 h were assessed with MTT assays. (**C**) Cell viability of Huh7^R^ cells treated with sorafenib, LY3214996, or the combination for 12 h, 24 h, and 48 h determined with MTT assays. (**D** and **E**) Colony formation assays were used to determine the clonogenic capacity of Huh7^R^ cells treated with sorafenib, LY3214996, or a combination of the two. Data are expressed as mean ± SD of three independent experiments (*p<0.05; **p<0.01; ***p<0.001).

To detect the inhibitory effects on Huh7^R^ cells treated with sorafenib, LY3214996 or a combination of the two, we measured cell viabilities by MTT assays. IC_50_s of sorafenib on Huh7 and Huh7^R^ cells were obtained (Fig. 2A). There was a dose- and time-dependent reduction in the cell viability of Huh7^R^ cells treated with sorafenib, LY3214996, and the combination of the two drugs (Fig. 2B and C). Compared with sorafenib or LY3214996 monotherapy, the combination had a stronger inhibiting effect on cell viability according to IC_50_ values. The results of colony formation assays also showed that sorafenib plus LY3214996 had stronger inhibitory effect on clone formation than did the control group and any single-agent treatment (Fig. 2D and E), which confirmed that LY3214996 enhanced the effects of sorafenib to suppress cell viability in Huh7^R^ cells.

### LY3214996 increased sorafenib inhibition of Huh7^R^ cellular migration

**Figure 3:**
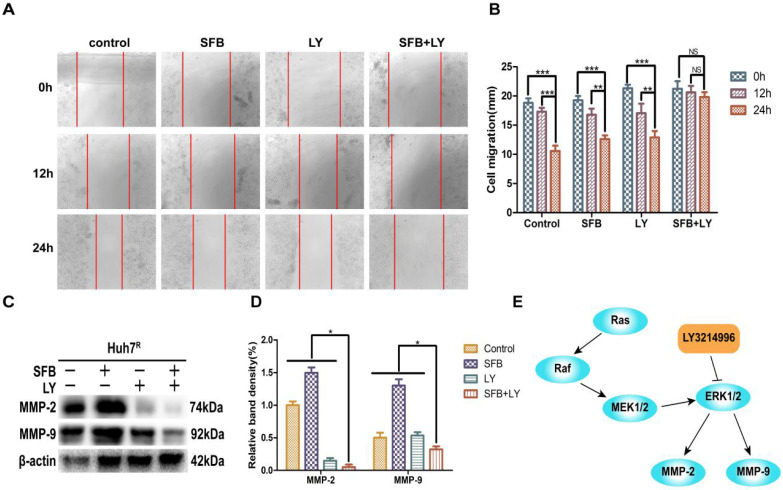
LY3214996 increased sorafenib inhibition of Huh7^R^ cell migration. (**A** and** B**) Wound healing assays were carried out to assess cell migration of Huh7^R^ cells treated with sorafenib, LY3214996, or the combination of the two for 12 h and 24 h. (**C** and **D**) Related proteins of tumor-cell migration (MMP-2 and MMP-9) were detected by western blot. (**E**) A proposed working model of how LY3214996 acts on the Ras/Raf/MAPK pathway to affect migration of Huh7^R^ cells. Data are expressed as mean ± SD of three independent experiments (* *p* < 0.05; **p < 0.01; ***p < 0.001).

To verify whether LY3214996 enhanced the inhibitory effect of sorafenib on Huh7^R^ cell migration, Huh7^R^ cells were treated with sorafenib, LY3214996, or a combination of the two for 12 h and 24 h, using the wound healing test. Compared to the control group, the wound size of cells treated with sorafenib or LY3214996 were 92.4% and 72.1% at 12 h and 67.3% and 61.4% at 24 h, respectively (*p*<0.05). The migration inhibition rates of LY3214996 combined with sorafenib were 94.1% at 12 h and 93.9% at 24 h (Fig. 3A and B), which was significantly higher than that of either single agent group (*p*<0.05). Western blot results revealed that after treatment with sorafenib combined with LY3214996, the expressions of MMP-2 and MMP-9 in Huh7^R^ cells were significantly lower than with monotherapy treatment (Fig. 3C and D) (*p*<0.05). These results indicated that the LY3214996 increased sorafenib inhibition of Huh7^R^ cell migration.

### LY3214996 combined with sorafenib induced cell-cycle arrest in the G0/G1 phase

**Figure 4:**
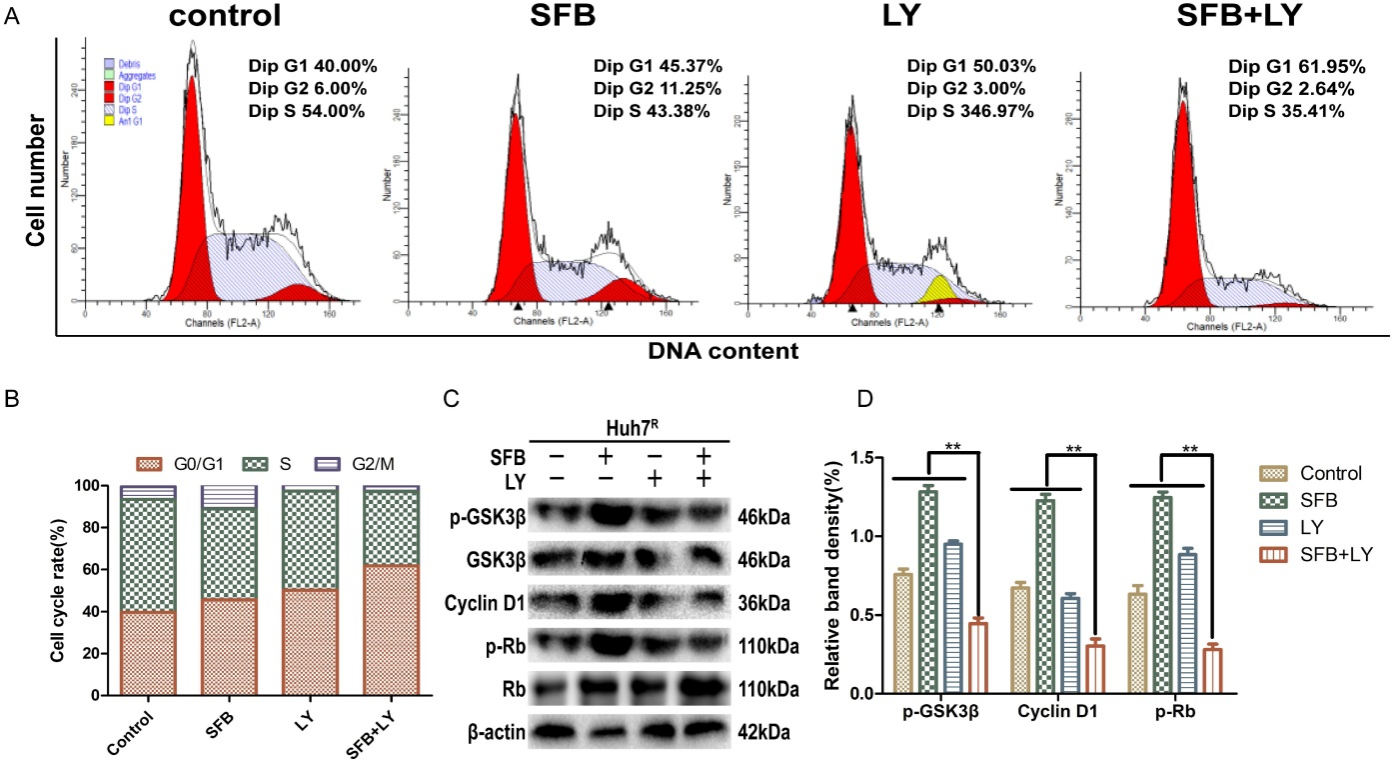
LY3214996 combined with sorafenib induced cell-cycle arrest in the G0/G1 phase. (**A**) and (**B**) Huh7^R^ cells accumulated at the G0/G1 phase after exposure to sorafenib (12 µM), LY3214996(1 µM), or the combination (sorafenib 12 µM, LY3214996 1 µM) for 24 h were measured by flow cytometry. (**C**) and (**D**) Cell- cycle protein expression (p-GSK3β, cyclin D1, and p-Rb) were quantified with western blot. Data expressed as mean ± SD of three independent experiments (*p<0.05; **p<0.01; ***p<0.001).

Abnormal cell-cycle progression causes unrestricted cell division, leading to continuous proliferation, which is a key driver of carcinogenesis 24. Thus, we assessed the effects of each treatment on the cell-cycle distribution by flow cytometry. After treatment for 24 h, the G0/G1 phase cell number percentages of cells treated with sorafenib plus LY3214996 (62.0%) (*p*<0.05) were more than with sorafenib (45.4%) or LY3214996 alone (50.0%) and the control group (40.0%) (Fig. 4A and B). Moreover, the combined treatment significantly increased the percentage of sub-G1 phase cells compared with the percentage in cells treated with either drug alone, which is evidence that the combination of two the drugs inhibited cell proliferation and promoted apoptosis.

Western blot results showed that the expression of cyclin D1 and p-Rb, two of the proteins that promote cell-cycle progression from G1 to S phase, were suppressed in LY3214996-treated Huh7^R^ cells. Furthermore, sorafenib combined with LY3214996 treatment led to decreased levels of cyclin D1 and p-Rb in the Huh7^R^ cells. Besides, the expression level of upstream protein p-GSK-3β was decreased after the combined treatment (Fig. 4C and D). These data indicate that combined sorafenib and LY3214996 treatment resulted in a pronounced cell-cycle arrest G0/G1 phase.

### LY3214996 increased sorafenib-induced apoptosis in Huh7^R^ cells

**Figure 5:**
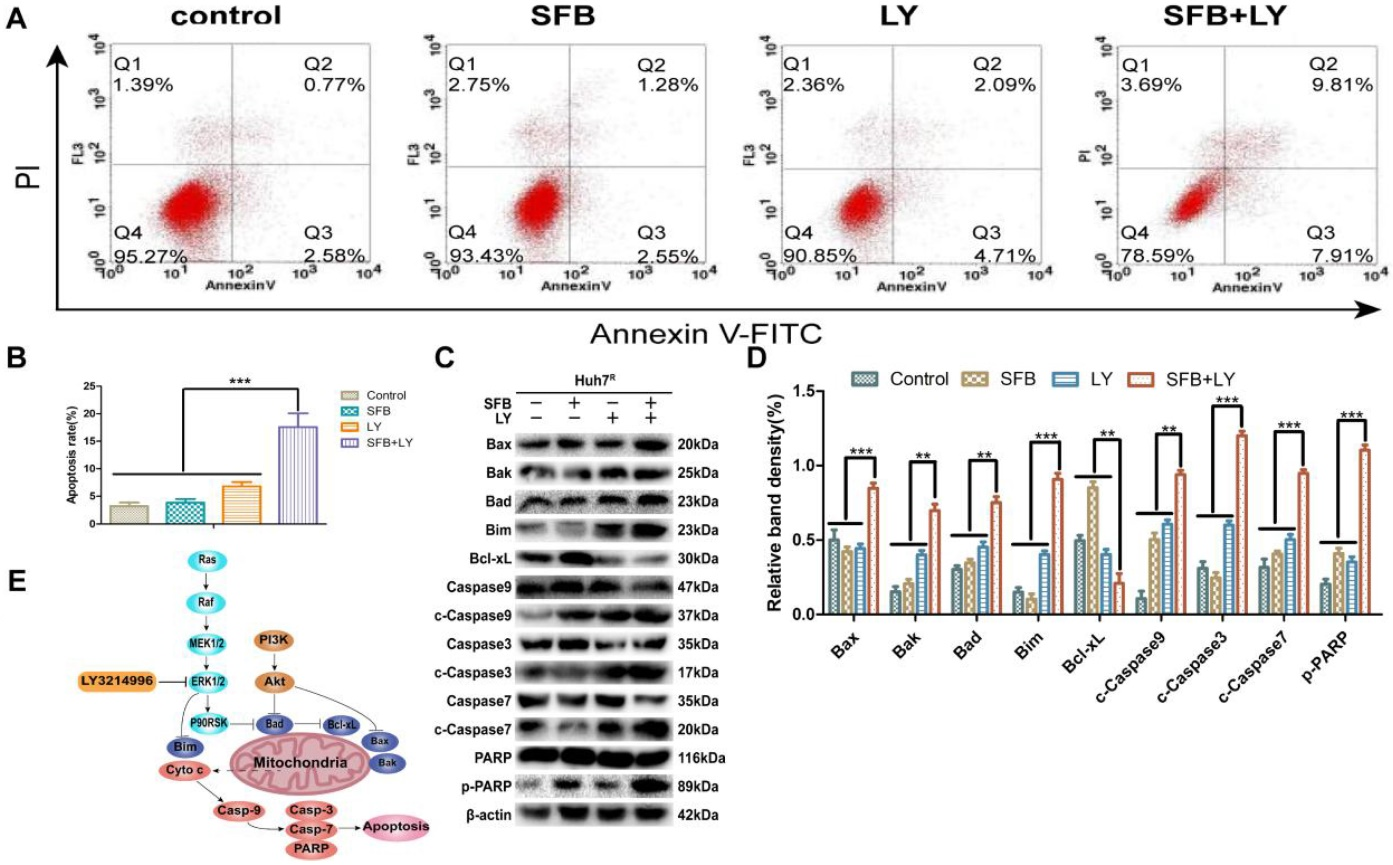
LY3214996 increased sorafenib-induced apoptosis in Huh7^R^ cells. (**A**) and (**B**) Apoptosis-related protein expression of Bcl-xL, Bax, Bak, Bad, Bim, caspase-3, -7, -9, and PARP were measured with western blot. (**C**) and (**D**) Cells were treated with sorafenib (12 µM), LY3214996 (1 µM), or in combination for 24 h. Apoptosis of Huh7^R^ cells was detected by flow cytometry with annexin V-FITC/propidium iodide staining. (**E**) Signal mechanism model of LY3214996 inducing mitochondrial apoptosis in Huh7^R^ cells. Data expressed as mean ± SD of three independent experiments (*p<0.05; **p<0.01; ***p<0.001).

To investigate the effect of combination treatment on Huh7^R^ apoptosis, Huh7^R^ cells were incubated with sorafenib (12 µM), LY3214996 (1 µM), or the combination, and the cells were harvested after 24 h and analyzed with flow cytometry. As illustrated in Figure 5, compared with the control group, the apoptosis rates of cells treated with sorafenib or LY3214996 were 5.8% and 11% (*p*<0.05), respectively; the apoptosis rate of cells treated with combined drugs was 17.7%, which was higher than with either single agent or control values (Fig. 5A and B) (*p*<0.05).

Immunoblotting analysis was performed to assay for apoptosis-related molecules to help understand the mechanism of the mitochondrial apoptotic pathway. The results revealed that levels of anti-apoptotic protein (Bcl-xL) in the combined treatment group were significantly down-regulated (*p*<0.05), while the key death-effector proteins (Bax and Bak), BH3-only proteins (Bad, Bim), and downstream proteins (caspase-3, -7, -9 and PARP) were significantly increased (Fig. 5C and D). These results suggest that sorafenib has a pro-apoptotic effect on Huh7^R^ cells when co-treated with LY3214996.

### LY3214996 enhanced anti-tumor effect by blocking Ras/Raf/MAPK pathway in Huh7^R^ cells

**Figure 6:**
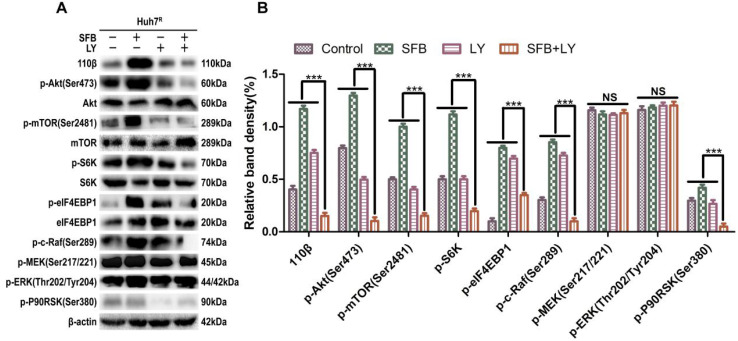
LY3214996 increases the anti-tumor effect by blocking Ras/Raf/MAPK pathway in Huh7^R^ cells. (**A**) and (**B**) After incubation with sorafenib (12 µM), LY3214996 (1 µM), or combination for 24 h, cells were lysed, and the designated proteins of Ras/Raf/MAPK and PI3K/Akt pathways were detected by western blot analysis. Data expressed as mean ± SD of three independent experiments (*p<0.05; **p<0.01; ***p<0.001).

Next, we explored further the anti-tumor mechanism of LY3214996 combined with sorafenib in Huh7^R^ cells. Western blot was performed to detect the levels of the major proteins, Ras/Raf/MAPK, in Huh7^R^ cells treated with LY3214996 or sorafenib monotherapy or in combination for 24 h. The results revealed that sorafenib treatment increased the values of p-Akt, p-mTOR, p-c-Raf, p-ERK1/2, p-S6K, p-eIF4EBP1, and p-P90RSK compared with values in the control group. However, the combination of sorafenib and LY3214996 led to suppression of p-Akt, p-mTOR, p-S6K, p-c-Raf, and p-P90RSK compared with control or sorafenib and LY3214996 treatment alone. Interestingly, p-ERK1/2 and p-MEK1/2 had an opposite result (Fig. 6A and B). Reactivation of p-ERK1/2 mediated by LY3214996 may be due to inhibition of p-c-Raf (S289/S296/S301), which are phosphorylation sites that inhibit feedback activation of ERK, an effect that suggests that the increase in p-ERK1/2 is due to the activation of c-Raf 25. These results indicate that LY3214996 enhanced anti-tumor effect by blocking the Ras/Raf/MAPK pathway and interfering with crosstalk of the PI3K/Akt pathway.

## Discussion

Chemotherapy is an important option for patients with advanced HCC, even though the available chemotherapeutic drugs have limited benefits and are accompanied by side effects 26. Sorafenib, as an oral multi-targeted kinase inhibitor, is well tolerated and significantly prolongs patient survival, which makes it the first choice for patients with advanced HCC 27. However, in many clinical studies and in practice, acquired resistance to sorafenib has resulted in a low response rate and limited clinical efficacy after a few treatment cycles 28. Therefore, it is necessary to explore agents that can overcome the resistance and improve the anti-tumor effect of sorafenib.

Activation of the PI3K/Akt pathway is highly related to the resistance of HCC to sorafenib 29-30. Sorafenib exerts its anti-tumor effect mainly by inhibiting the Ras/Raf/MAPK pathway, but crosstalk activates the PI3K/Akt pathway during this process 31. Despite feedback activation of p-ERK1/2, LY3214996 still inhibits the downstream signals of ERK signaling 32. In this study, we found that the levels of p-Akt and p-ERK1/2 in Huh7^R^ cells were higher than in Huh7 cells, while p-Akt and p-P90RSK were downregulated by a combination treatment of LY3214996 and sorafenib. This result suggested that that the PI3K/Akt pathway was activated with sorafenib treatment, and inhibited ERK1/2 could suppress Akt activation. Due to ERK or RSK activation directly mediating the PI3K/Akt/mTOR pathway 33-34, leading to compensated proliferation and anti-apoptotic effects, blocking the up-regulation of PI3K/Akt/mTOR pathway induced by ERK activation is an effective strategy to decrease sorafenib resistance and improve efficacy of the drug.

Some studies have shown that sorafenib-resistant HCC cells have enhanced migration and invasion ability by activating epithelial-mesenchymal transition after long-term sorafenib treatment 35-36. MMP2 and MMP9 participate in angiogenesis by destroying basal-layer molecules and remodeling the extracellular matrix during angiogenesis 37. The MMPs in the extracellular matrix participate in the acquisition of migration characteristics and make it easier for tumor cells to invade surrounding tissues and metastasize to secondary sites 38. It has been reported that ERK1/2 can regulate MMP-2 and MMP-9 expression 39. Similar results were found in our study, and when Huh7^R^ cells were exposed to the combination of LY3214996 and sorafenib, the wound size in wound-healing assays was significantly more than with sorafenib or LY3214996 monotherapy, and the expression levels of MMP-2 and MMP-9 were correspondingly reduced.

PI3K/Akt signaling is closely related to the mitochondrial apoptosis pathway. P-Akt phosphorylated Bad, a member of the Bcl-2 family, which was followed by Bcl-xL induced anti-apoptosis 40. LY3214996 plus sorafenib treatment inhibited p-Akt and p-P90RSK but upregulated Bad and Bim and induced a cascade of mitochondrial apoptotic proteins, caspase-9, caspase-3, and caspase-7. Thus, sorafenib and LY3214996 inhibited the Ras/Raf/MAPK and PI3K/Akt pathways, leading to activation of the mitochondrial apoptotic pathway. LY3214996 evidently enhanced the anti-tumor effect of sorafenib by inhibiting the Ras/Raf/MAPK pathway to promote the mitochondrial apoptotic pathway.

LY3214996 has induced G1 cell-cycle arrest in cell lines of melanoma, colorectal cancer, pancreatic cancer, and non-small cell lung cancer 32. Our results revealed that combined treatment of LY3214996 and sorafenib suppressed the expression of cyclin D1 and p-Rb, then increased the proportion of cells in the G0/G1 phase to enhance the anti-proliferative effect of sorafenib on Huh7^R^ cells, as others have reported also 28.

Overall, we found that acquired resistance to sorafenib in Huh7^R^ cells resulted in limited effectiveness in treatment. One of the most important mechanisms of this result was the activation of PI3K/Akt and Ras/Raf/MAPK pathways. Inhibition of ERK simultaneously blocked the downstream MAPK pathway and crosstalk with PI3K/Akt, which provides a theoretical basis for overcoming sorafenib resistance in patients with advanced HCC. Since the experiment was limited to *in vitro* studies, our future work will focus on *in vivo* research, and more signaling pathways will be assessed. We believe that sorafenib combined with LY3214996 can be a promising strategy in the treatment of sorafenib-resistant HCC.

## Conclusions

The present study demonstrated that PI3K/Akt and MAPK/ERK pathways are abnormally activated in sorafenib-resistant HCC cells. Sorafenib combined with LY3214996 inhibited these two pathways to enhance anti-tumor effects by inducing apoptosis and inhibiting cell proliferation. These findings form a theoretical basis for trial of LY3214996 combined with sorafenib as second-line treatment of sorafenib-resistant, advanced HCC.

## Abbreviations

HCC: hepatocellular carcinoma
SFB: sorafenib
MEK: mitogen-activated protein kinase
ERK: extracellular signal-regulated kinase
HRP: horseradish peroxidase
PBS: phosphate buffered saline
FITC: fluorescein isothiocyanate
SDS-PAGE: sodium dodecyl sulfate-polyacrylamide gel electrophoresis
